# Oestrogens and oestrogen receptors in prostate cancer

**DOI:** 10.1186/s40064-016-2185-6

**Published:** 2016-04-26

**Authors:** Karolina Kowalska, Agnieszka Wanda Piastowska-Ciesielska

**Affiliations:** Department of Comparative Endocrinology, Faculty of Biomedical Sciences and Postgraduate Training, Medical University of Lodz, Zeligowskiego 7/9, 90-752 Lodz, Poland

**Keywords:** Oestrogen, Oestrogen receptor α, Oestrogen receptor β, Prostate cancer

## Abstract

The role of androgens in prostate cancer is obvious due to the fact that androgen signalling is the main regulator of prostate growth and function. Androgen deprivation therapy is a mainstay treatment for advanced prostate cancer. However, prostate cancer often becomes androgen-independent, which in consequence leads to lethal and incurable disease. In addition, oestrogens play a crucial role in prostate cancer, especially in elder men in whom the overall ratio of oestrogens to androgens is increasing. This review summarizes the current knowledge on molecular mechanisms through which oestrogens are involved in prostate cancer development. We focused on commonly alternated molecular signalling pathways contributing to tumourgenesis in prostate cancer.

## Prostate cancer

Prostate cancer (PCa) is one of the most common malignance in men in developing countries. In Western countries it is the second cause of death in men, and in the majority of cases it is associated with metastases (Bishop et al. 2015). In recent years the incidences of prostate cancer have been increasing with simultaneous increase in survival rate of the diagnosed patients (Wojciechowska et al. 2015). The progress made in treatment of this disease is probably associated with detection of prostate-specific antigen (PSA) which is currently used as a prognostic marker detected in patients’ serum (Stelmach et al. 2011). Although many research studies have been carried out on prostate cancer, detailed molecular mechanism has not been revealed. It is known that age, race, ethnicity or place of living might be causative factors for prostate cancerogenesis. Also diet and lifestyle are recognized as carcinogenic factors (Mahmoud et al. 2015). PCa could be classified on the basis of different morphologic types, of which the most common is adenocarcinoma derived from epithelium, neuroendocrine tumours, sarcomas and lymphomas (Wojciechowska et al. 2015). Treatment of the early stages of PCa are usually focused on deprivation of androgens, known as: androgen deprivation therapy (ADT). In advanced stages prostate cancer cells begin to be androgen independent and present higher metastatic potential (Suva et al. 2011).

## Oestrogens and oestrogens receptors

Oestrogens play a crucial role in human development, maintaining sexual and reproductive functions of the organism, as well as influencing the function of cardiovascular, immune, skeletal and central nervous system. The most potent oestrogen produced by the body is 17β-estradiol (E2) (Heldring et al. 2007). Nevertheless, clinical evidence obtained by Huggins and Hodges showed that oestrogens can influence prostate cancer by inhibiting tumour growth (Huggins and Hodges 1972). Nowadays, oestrogens are used in prostate cancer therapy to reduce follicle-stimulating hormone (FSH) production and decrease stimulation of hypothalamic pituitary by luteinizing hormone (LH), which in consequence reduces androgen synthesis. Although the usage of oestrogens in PCa therapy seems to be favourable, castrate-resistant prostate cancer cells (CRPC) can overcome the mechanisms mentioned above and progress in the disease. Moreover, oestrogen therapy has numerous cardiovascular and thrombotic side effects that reduce its clinical use as an alternative to castration (Christoforou et al. 2014).

**Fig. 1:**
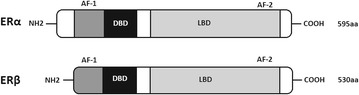
Schematic representation of oestrogen receptors isoforms: α (ERα) and β (ERβ). AF-1 and AF-2: distinct transcription activation sites; DBD—DNA-binding domain; LBD—ligand-binding domain; NH_2_– and –COOH are terminal regions of protein; ERα is 595 amino acids length, ERβ 530 amino acids length

Cellular signalling of oestrogens is triggered by oestrogen receptors (ERs) α (ERα) and β (ERβ) which are the members of nuclear receptor superfamily (NR) of transcription factors (Christoforou et al. 2014). In prostate ERβ is present in epithelial cells, whereas ERα in stromal cells (Powell et al. 2012). ERs contain evolutionary conserved structurally and functionally distinct domains, characteristic for the family of nuclear receptors (Fig. 1). DNA-binding domain (DBD) involved in DNA recognition is the central and the most conservative domain. Ligand binding takes part at COOH-terminal multifunctional ligand-binding domain (LBD). The most variable domain is NH_2_-terminal domain which is also not conserved. Two distinct activation sites: AF-1 and AF-2 enable transcriptional activation (Heldring et al. 2007).

**Fig. 2:**
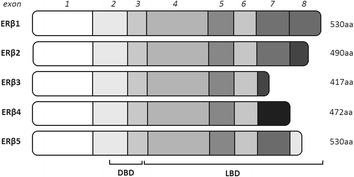
Schematic structure of oestrogen receptor β (ERβ) isoforms. First six exons are the same in different isoforms. All isoforms share the same DNA-binding domain (DBD) but differ in ligand-binding domains (LBD). On the right side the length of amino acids products is presented

ERα and ERβ are products of genes localized on different chromosomes. They have the ability to dimerize with full length ERα and repress AF-1 mediated activity (Wang et al. 2005). In humans five forms of ERβ were identified ERβ1, ERβ2, ERβ4 and ERβ5 in normal prostate cell (Fig. 2; Santamaria-Martinez et al. 2008). Recently, it was suggested that only ERβ1 isoform may be fully functional, due to the fact that it is the only isoform which may form homodimers or take part in recruitment of coregulatory proteins (Leung et al. 2006). More attention should be placed on ERβ2 and ERβ5 isoforms. They have the same sequence as ERβ1 from exons 1–7 and contain extra sequences with lost AF-2 domain function (Leung et al. 2010). Moreover they can form heterodimers with ERβ1 isoform after oestrogen stimulation (Leung et al. 2006) or with ERα and through this silence signalling of ERα (Ogawa et al. 1998).

In recent years more attention is paid to oestrogen receptors in prostate cancer indicating that ERα and ERβ may play an opposite role in prostate cancer. Firstly, it was suggested that ERα stimulates cell proliferation and transcription and has opposing role to ERβ whose expression is lost during the progression of the disease (Fixemer et al. 2003). Currently, the antiproliferative role of ERβ is not so obvious due to research presenting that ERβ2 and ERβ5 isoforms have tumour-promoting roles (Nelson et al. 2014). ERα-knockout mice do not develop prostate cancer after stimulation of testosterone or oestrogens, whereas ERβ-knockout mice do, similarly to the wild type mice (Ricke et al. 2008). Furthermore, ERβ1 might complex with AR and cause a transcription of AR-related genes in PCa, what might explain the fact of elevated ERβ expression in HNPC, corresponding to lower survival of patients (Nelson et al. 2014). Nevertheless, many research studies have been conducted to reveal the detailed molecular mechanism of oestrogen receptors function in PCa.

In this article we reviewed the molecular signalling pathways in which oestrogen receptors are involved in prostate cancer cells (Table 1): cell proliferation and apoptosis as the two pathways mainly altered in carcinogenesis by gene mutations or improper regulation of gene expression (Dey et al. 2012) what can be confirmed by the fact that in human prostate tumours an increase in apoptosis and simultaneous decrease in proliferation are found 1–7 days after castration and retrieval of these markers in some patients is possible after 10 days (Ohlson et al. 2005); then epithelial to mesenchymal transition (EMT) and cell invasiveness as the two processes mainly responsible for metastases which enables prostate cancer cells to engraft in the bone marrow places, as well as the fact that the molecular crosstalk between the prostate tumour and bone stroma in which EMT genes participate is a target of prostate cancer therapies (Smith and Bhowmick 2016); and the chronic inflammation frequently detected in prostate biopsies in patients with increased prostate specific antigen (PSA). Moreover, it was showed that chronic inflammation in benign prostate tissue contributes to high-grade prostate cancer and novel biomarkers for predicting tumour progression due to prostatic inflammation in prostate cancer patients were investigated (Stark et al. 2015).

**Table 1:** Summarized biochemical pathways and genes involved in oestrogen signalling in prostate cancer

Gene/signaling pathway		Oestrogen receptor type participating in action	Author (year)
*Cell proliferation*			
Oncogenes	BMI-1	ERβ	Mak et al. (2006)
	Twist	ERβ2	Dey et al. (2012)
Suppressors	PTEN	ERα	Takizawa et al. (2015)
	MAPK	ERα	Takizawa et al. (2015)
Cell cycle control	p21	ERβ	Colciago et al. (2014)
	CCND1	ERβ	Nakamura et al. (2013)
	c-Myc	ERβ2	Dey et al. (2012)
Signaling pathways	PI3 K	ERα	Takizawa et al. (2015)
	mTOR	ERα and ERβ	Wang et al. (2014)
*Apoptosis*			
Proapoptotic	Bax	ERβ	Cheng et al. (2004)
	Cleaved caspase-3	ERβ	Cheng et al. (2004)
	p-53	ERs	Kanagaraj et al. (2007)
	FOXO3 and PUMA	ERβ	Dey et al. (2014)
Pro/antiapoptotic	TGF-β1	ERs	Chipuk et al. (2001)
*Epithelial*-*mesenchymal transition (EMT)*			
↓EMT	PHD2/EGLN1	ERβ1	Mak et al. (2006, 2010)
	HIF1-α	ERβ1	Mak et al. (2006, 2010)
	Snail1	ERβ1	Mak et al. (2006, 2010)
	Runx 2	ERβ2	Dey et al. (2012)
	TGF-β1	ERβ	Hu et al. (2015)
	E-cadherin	ERβ	Hu et al. 2015
	Thbs2	ERα	Slavin et al. (2014)
	MMP3		Slavin et al. (2014)
↑EMT	SOX4	ERβ	Mak et al. (2010)
	Twist1	ERβ2	Dey et al. (2012)
	c-Myc	ERβ2	Dey et al. (2012)
	HIF-1α	ERβ2 and ERβ5	Dey et al. (2015)
	Runx 2	ERβ1	Dey et al. (2012)
	NEAT-1	ERβ	Chakravarty et al. (2014)
*Chronic inflammation*			
↑	HIF-1α	ERα	Ravenna et al. (2009, 2014)
	NF-κB	ERα	Ravenna et al. (2009, 2014)

## Prostate cancer cell proliferation

**Fig. 3:**
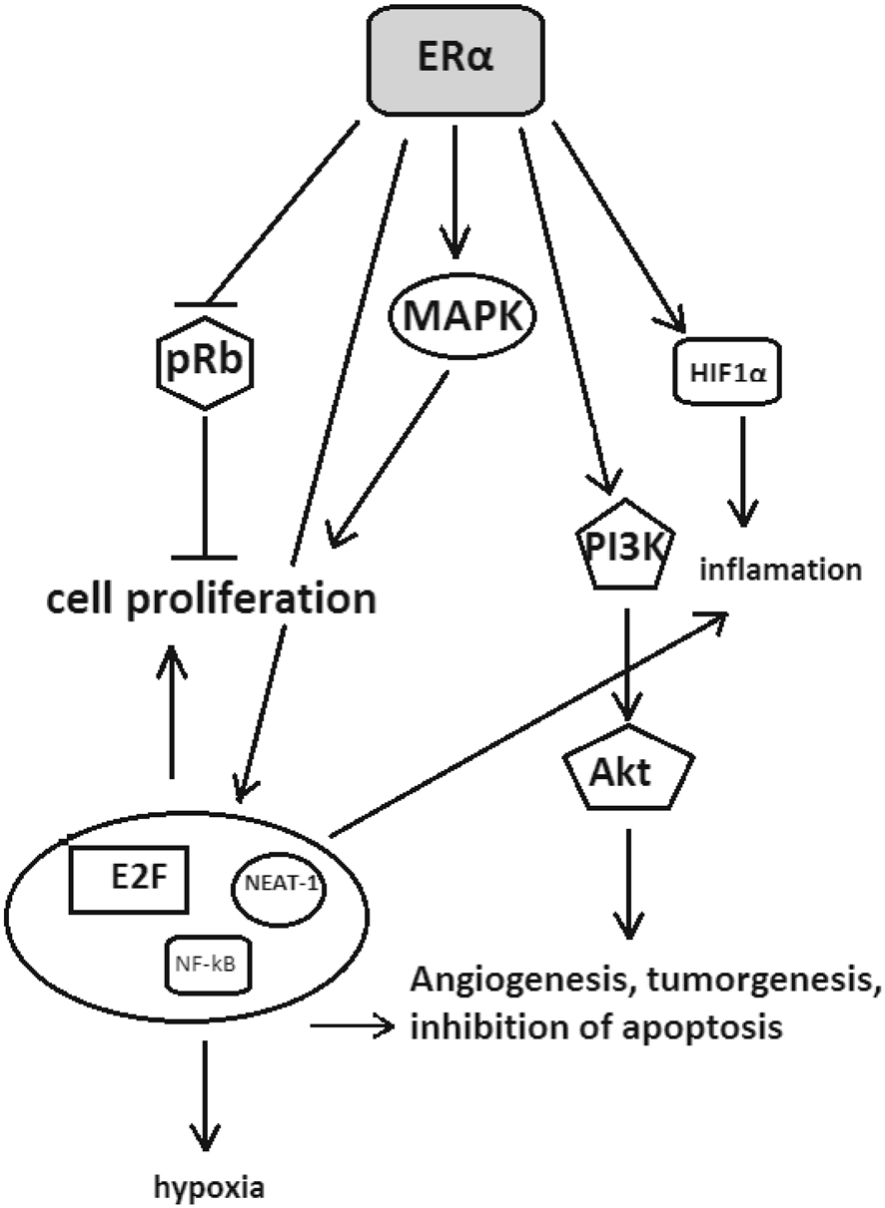
Summarized cell signalling pathways in which oestrogen receptor α (ERα) takes part (detailed description presented in the text)

The role of ERα in prostate cancer cell proliferation is well documented (Fig. 3). Mice lacking ERα did not present aggressive tumour phenotype, contradictory to mice lacking ERβ (Slusarz et al. 2012). PTEN-deficient mice which are used in prostate cancer studies showed significantly increased expression of ERα in regions were cell proliferation was elevated. Depletion of ERα caused a significant decrease in the size of prostate cancer cell colonies without changing their number. Mitogen-activated protein kinases (MAPK) activity and phosphoinositide 3-kinase (PI3K) signalling pathways are sustained by ERα in prostate cancer (Takizawa et al. 2015). Fujimura et al. (2014) found a relationship between biochemical recurrence, prognoses and ERα expression. ERα counterparts in oestrogen induced translocation of prohibitin (PHB) known to have a significant effect on cell senescence and tumour cell suppression through modulation of retinoblastoma protein (pRB) binding with transcription factors family (E2F) complex which leads to repression of pRb-E2F-associated transcription and cell proliferation. Silencing ERα using siRNA approach was able to suppress PC3 (human prostate cancer adenocarcinoma cell line) cells proliferation and translocation of PHB from mitochondria to the nucleus (Dong et al. 2013). Hariri et al. (2015) have recently shown that low doses of toremifene, a ERα blocker, causes necrosis with high expression of ERα on the stromal surface of the PC3M (cell line derived from a liver metastasis of PC-3 intraspleenic injection in nude mice) cancer cells.

Many research studies are focused on detailed molecular mechanism of ERβ action in prostate cancer. Moreover, it seems that antiproliferative function of all ERβ isoforms is not so obvious. *In vitro* models of prostate adenocarcinoma expressing only ERβ showed that stimulation of estradiol caused significant reduction of cell proliferation (Corey et al. 2002). Interestingly, cell transfection with ERβ1 or ERβ2 revealed that only ERβ1 isoform inhibits proliferation of prostate cancer cells (Dey et al. 2012). Cell cycle genes play an important role in the control of cell proliferation and tumourgenesis. Cyclin D1 (CCND1) is overexpressed in many types of human cancer including prostate cancer. Interaction between androgens and cell cycle associated proteins was reported by Perry and Tindall in 1996. Like androgens, oestrogens have a capability to modulate cell cycle progression. ER inhibitor ICI 182,780 was reported to influence CCND1 expression and modulate progression to late G1 cell cycle phase (Santamaria-Martinez et al. 2008). The studies by Nakemura et al. (2013) revealed that oestrogens modulate CCND1 expression trough ERβ via increasing FBJ murine osteosarcoma viral oncogene homolog (FOS) and jun proto-oncogene (JUN) expression in prostate adenocarcinoma PC3 cell line. Cyclin D1-mediated cell cycle pathway might be also modulated by serum/glucocorticoid regulated kinase family member 3 (SGK3)-androgen receptor (AR) expression. Wang et al. documented that in AR-positive cells both ER receptors are involved in regulation of cell cycle through mammalian target of rapamycin (mTOR) signalling pathway (Wang et al. 2014). Nuclear ERβ2 isoform was reported to have an oncogenic role due to its ability to increase proliferation and expression of twist family bHLH transcription factor 1 (Twist1) and v-myc avian myelocytomatosis viral oncogene homolog (c-Myc) in PC3 and 22Rv1 carcinoma human prostate cancer cell lines (Dey et al. 2012). Endogenous activation of ERβ in PC3 and DU-145 (prostate carcinoma derived from metastatic site—brain) cells was reported to cause an increase in cyclin-dependent kinase inhibitor 1A (p21) expression and cell cycle arrest indicating that control of proliferation might be governed by cell cycle modulation (Colciago et al. 2014).

In males ERβ ligand 3β-Adiol which is a product of 5α-reduction of testosterone to 5α-dihydrotestosteron (DHT), is present in high levels. Its binding to ERβ receptor in prostate cancer cells is sufficient to inhibit PCa proliferation with simultaneous inhibition of migration and invasiveness of PCa cells (Dondi et al. 2010). Another molecular mechanism of cell cycle modulation caused by ERβ was proposed by Mak et al. They postulated that ERβ is repressed in prostate cancer through polycomb complex protein BMI-1 (BMI-1), which is induced by phosphatase and tensin homolog **(**PTEN) deletion. BMI-1 is a well-known oncogene regulating cell proliferation and senescence in prostate tumourgenesis (Mak et al. 2006).

## Apoptosis of prostate cancer cells

**Fig. 4:**
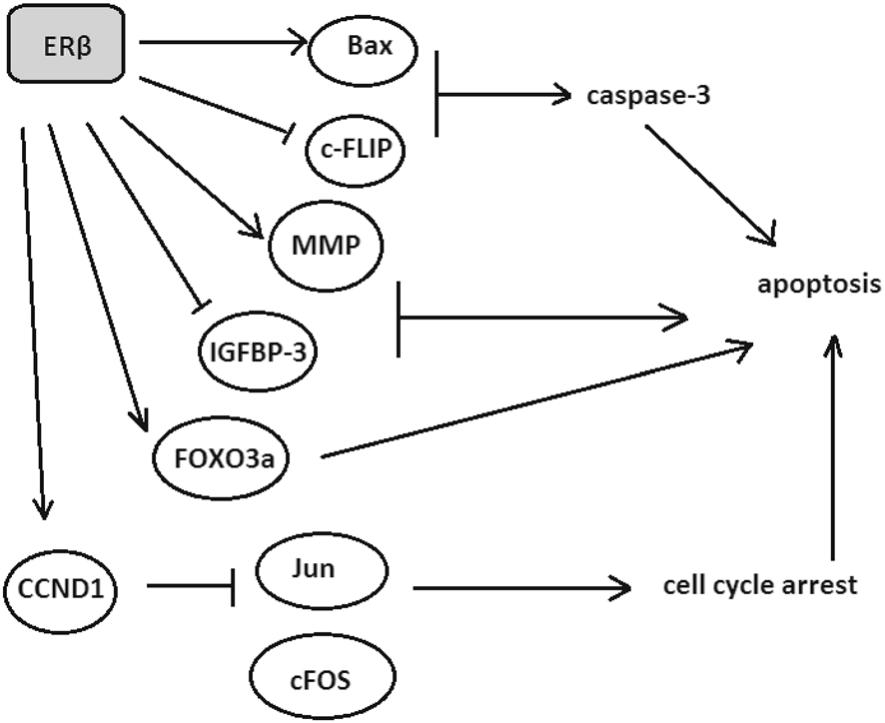
Summarized cell signalling pathways in which oestrogen receptor β (ERβ) induces apoptosis in prostate cancer. ERβ through different pathways induces cell cycle arrest and apoptosis (detailed description is presented above in the text)

Aside from the role of ERβ in inhibiting PCa cell proliferation and growth, many studies have shown that this oestrogen receptor may be involved in regulation of programmed cell death (Fig. 4). Cheng et al. (2004) suggested that ERβ is a stronger regulator of cell growth that ERα in prostate cancer. Moreover, they presented that introduction of ERβ to DU-145 cell line led to a strong increase in pro-apoptotic protein BCL2-associated X protein (Bax) and peri-nuclear expression of cleaved caspase-3. ERβ might also downregulate c-FLIP (cellular FLICE (FADD-like IL-1β-converting enzyme)-inhibitory protein) promoter by binding to Sp1 site or by modulation of Sp1/Sp3 ratio through competitive DNA binding, and then promote apoptosis in prostate cells (Yun et al. 2015). Miro et al. (2011) suggested that oxidative stress might be a causative factor in apoptosis. The effect of β-estradiol on prostate cells in the case of oxidative stress is dependent on ERα/ERβ ratio and might cause an increase in mitochondrial production of reactive-oxygen species (ROS) by repressing the uncoupling proteins (UCPs), which is consistent with previously reported ERα pro- and ERβ antioncogenic effect. UCPs are involved in the maintenance of mitochondrial membrane potential and ROS production (Valle et al. 2010). It was shown that prolonged increase in Ca^2+^ concentration may lead to apoptosis. 17β-estradiol through ERs can cause a concentration-dependent increase in Ca^2+^ concentration in PC3 cells, thus modulate the activity of Ca^2+^-dependent enzymes and tumourgenesis of prostate tissue (Huang and Jan 2001). Kanagaraj et al. (2007) found that estradiol causes apoptosis in PC3 cells due to reduced matrix metalloproteinase (MMP) level and increased Insulin-like growth factor-binding protein 3 (IGFBP-3), which was reported to induce apoptosis through tumour protein p53 (p53)-dependent manner. Apoptosis in prostate cancer might be also triggered by ERβ-induced increase in transcription of forkhead box O3 (FOXO3a)-transcription factor known as a tumour suppressor. Increased FOXO3a transcription then causes an increase in PUMA expression (a pro-apoptotic factor p53-upregulated modulator of apoptosis) and triggers apoptosis in prostate cancer cells (Dey et al. 2014). In epithelial prostate cells TGF-β1 has been shown to induce apoptosis via release of cytochrome c (Chipuk et al. 2001). There is also evidence presenting that during tumour progression the inhibitory effect of TGF-β1 may promote tumour growth via its angiogenic effect. Brodin et al. (1999) suggested that activation of SMAD family member 2 (Smad2) and upregulation of SMAD family member 3, 4, 6 and 7 (Snad3, Smad4, Smad6 and Smad7) are associated with induction of apoptosis in both normal and cancer prostate cells.

## EMT and cell invasiveness

**Fig. 5:**
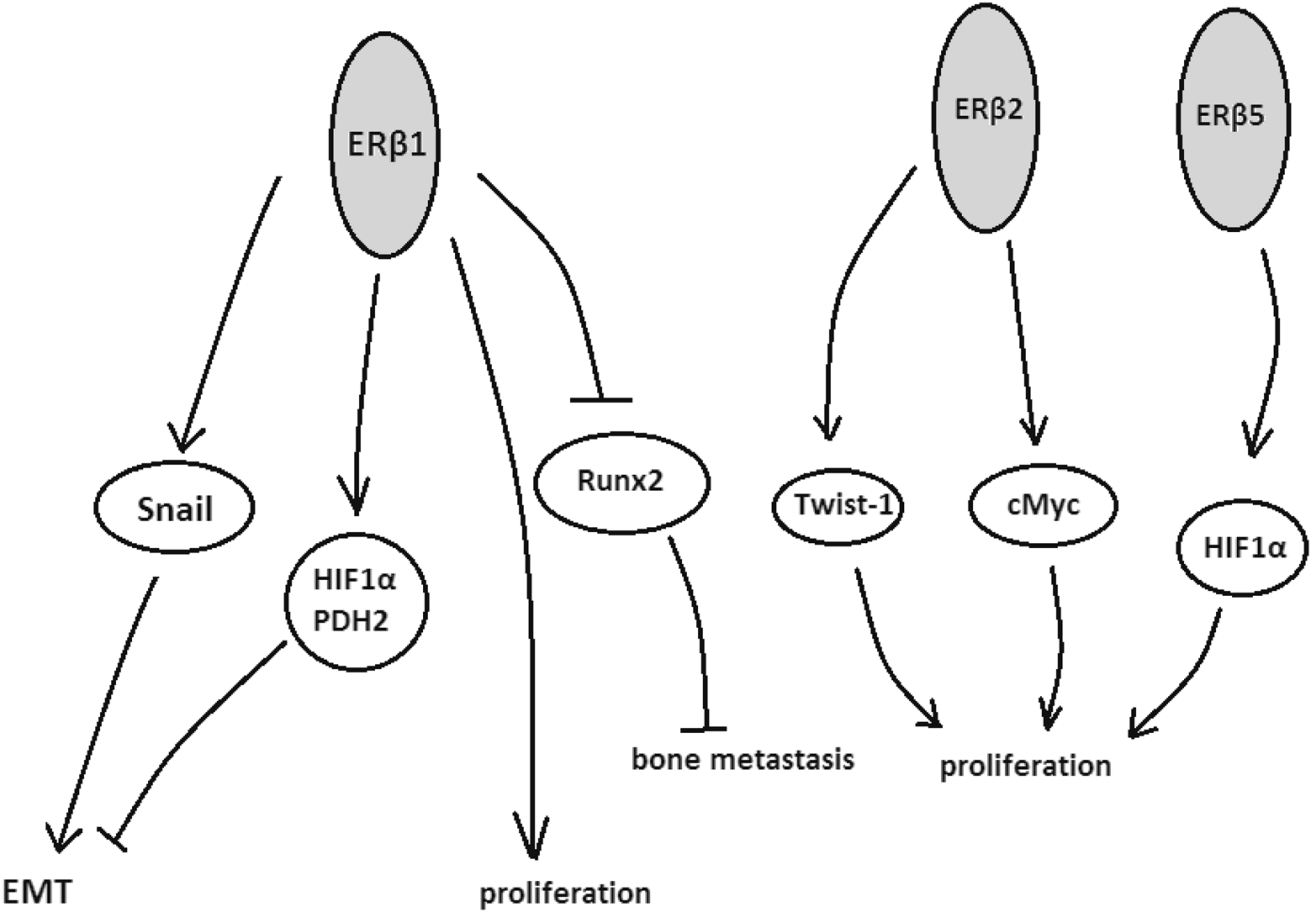
Schematic cell signalling pathways in which different isoforms of oestrogen receptor β (ERβ) might be involved in dualistic action in prostate cancer. Different signalling pathways might be associated with different homo- and hetero-dimerization of ERβ isoforms (not presented in the figure). Detailed description presented above in the text

It is well known that high grade PCa lose their epithelial phenotype and show mesenchymal characteristics, such as expression of vimentin and vascular endothelial growth factor (VEGF), loss of E-cadherin and increase in expression of hypoxia-inducible factor 1α (HIF-1α) (Christoforou et al. 2014). The sex determing region Y-box (SOX4) is a transcription factor commonly overexpressed in PCa. Estradiol up-regulates SOX4 expression by formation of ERβ and AR protein complex. Moreover, clinical data reported in China suggested that overexpression of SOX4 is significantly associated with ERβ expression and EMT in patients (Yang et al. 2015). ERβ1 was shown to inhibit EMT by upregulating propyl hydroxylase domain 2 (PHD2/EGLN1) and decreasing hypoxia inducible factor 1 alpha subunit (HIF-1α) levels, as well as repressing transcription of vascular endothelial growth factor A *(VEGF*-*A)* (Fig. 5). Mak et al. (2010) suggested that ERβ1 regulates snail family zinc finger 1 (Snail1) via the regulation of glycogen synthase kinase 3 **(**GSK-3β) activity, an enzyme that is crucial for regulation of Snail1 localization and stability (Mak et al. 2006, 2010). On the other hand ERβ2 was reported to increase invasiveness of PCa cells trough expression of Twist1 and c-Myc (Dey et al. 2012). ERβ2 and ERβ5 were shown to stabilize HIF-1α protein and induce hypoxic gene expression indicating its oncogenic effect in PCa (Dey et al. 2015). In vitro studies on PC3 cell line demonstrated that ERβ1 was able to repress the expression of Runx2, which is known to be involved in bone metastases in prostate cancer, whereas ERβ2 isoforms triggered a contradictory effect (Dey et al. 2012). Slater et al. (2002) presented data suggesting that increase in ERβ receptor might be simultaneous with increase of E-cadherin expression and decrease of TGF-β1 in prostate epithelium. Similar effect was observed by Mak et al. (2010) who noted that TGFβ1 signalling decreased ERβ expression in both AR-dependent and AR-independent manner. Moreover, ERβ knock down using shRNA caused a significant increase in EMT (Mak et al. 2010). Another protein involved in EMT in prostate cancer might be periostin which is also known as osteoblast-specific factor 2. It was confirmed that periostin is induced by TGF-β/STAT3/Twist1 pathway and participates in TGF-β-induced EMT (Hu et al. 2015). Modulators of oestrogen receptors were shown to decrease cell migration and this effect was reversed by antioestrogens, indicating that anti-migratory action of phytoestrogens is directly associated with ERs (Piccolella et al. 2014). It is known that loss of ERβ may correlate with the development of poorly differentiated PCa (Slusarz et al. 2012). Mak et al. (2015) suggested a molecular mechanism associated with repression of ERβ in PCa. ERβ loss is caused by PTEN deletion, which is one of the most common genetic mutations in prostate cancer. ERβ transcription is diminished by BMI-1 which is induced by PTEN deletion. This mechanism cooperates with abovementioned HIF-1/VEGF signalling pathway altered in prostate cancer cells (Mak et al. 2015). Another mechanism associated with ERs in prostate cancer may involve interaction of ERs with nuclear paraspeckle assembly transcript 1 (NEAT-1), a transcriptional regulator which contributes to tumourgenesis. Chakravarty et al. (2014) showed that ERα transcriptionally regulates NEAT1 and promotes prostate tumour progression both in vitro and in vivo. Prostate cancer invasion might be suppressed by cancer-associated fibroblasts (CAFs) in which ERα was reported to modulate thrombospondin 2 (Thbs 2) and matrix metalloproteinase 3 (MMP-3). ERα in CAFs probably decreases invasiveness through decreasing tumour angiogenesis (Slavin et al. 2014).

## Chronic inflammation in prostate cancer

ERs might be also associated with inflammation in prostate cancer. Yatkin et al. (2009) suggested that reduced androgen/oestrogen ratio might induce inflammation in rat prostate. Harris et al. (2000) suggested that oestrogens play a proinflammatory role and high androgen concentration plays a protective role. They showed that 4 days oestrogen treatment caused an increase in interleukin-1 beta, 6 and inflammatory protein 2-alpha (IL-1β, IL-6, MIP-2) and inducible nitric oxide synthase (iNOS) levels in in vivo model. Moreover, oestrogens effect on cell inflammation is probably associated with ERα activity. Prins et al. (2001) observed that after treatment with oestrogens inflammation was present only in ERβ-knockout mice, but not in ERα-knockout mice. It was suggested that on the molecular level only *ERα* and epidermal growth factor receptor (*EGFR)* expression correlates with the levels of all proinflammatory molecules, and HIF1-α and nuclear factor kappa-light-chain-enhancer of activated B cells (NF-kB) are the master regulators of hypoxia and inflammation, respectively, in prostate cancer cell lines (Ravenna et al. 2009, 2014).

## Oestrogens in prostate cancer therapy

Androgen deprivation therapy (ADT) is recommended for the treatment of advance prostate cancer patients (Yu et al. 2015). Oestrogens were used as a gold standard in prostate cancer therapy years ago. Many research studies showed that oral oestrogens might increase cardiovascular toxicity which in association with the age of patients limits the usage of oestrogens caused by the effect of the first-pass hepatic metabolism on coagulation (Stein et al. 2012; Langley et al. 2013). Nevertheless, nowadays more attention is paid to oestrogens, due to the fact that gonadotropin-releasing hormone (GnRH) agonists used as a standard treatment have oestrogen-deficiency related side effects (Yu et al. 2015; Stein et al. 2012). It seems that avoidance of the oral administration might limits cardiovascular side effects associated with oestrogens. Thus, parenteral administration of oestrogens might be useful in prostate cancer therapy and might be an alternative to current standard therapy (Langley et al. 2013). The usage of diethylstilbestrol (DES) a synthetic oestrogen was reported to possess acceptable toxicity and might be considered for further clinical studies (Wilkins et al. 2012). Tormifene which belong to second-generation of selective oestrogen receptor modulators (SERMs) was reported to decrease the incidence of new cardiovascular side effects in men younger than 80 years old in ADT treated patients in 80 mg dose (Smith et al. 2011). On the other hand for the dose of tormifene 20 mg no beneficial effect was observed in another study (Taneja et al. 2013). Clinical significance was also established for 2-methoxyoestradiol (2ME2) which possesses anti-angiogenic and anti-proliferative characteristics for patients with CRPC with no beneficial effect observed (Harrison et al. 2011). Interestingly, its analogue ENMD-1198 was recommended for phase II clinical studies as beneficial for patients (Zhou et al. 2011). The agonist of ERα GTx-758 significantly decreased oestrogen deficiency side effects during ADT, although it increases the incidence of venous thromboembolism (VTE) (Yu et al. 2015). Thus, more clinical studies should be carried out to exclude potential side effects associated with known oestrogens or to determine the potential usage of the new ones.

## Conclusions

In recent years there is a growing body of evidence that oestrogens play crucial role in prostate tumourgenesis. Here we presented data showing that oestrogen, acting via its receptors, regulates various cellular processes including: proliferation, differentiation, apoptosis, EMT, invasiveness and chronic inflammation in prostate cancer cells. It seems that ERα possesses oncogenic role in prostate cancer, whereas ERβ suppressive role has been disputable so far. Now, many research studies have shown that ERβ isoforms might act differently in prostate cancer indicating that its role is pleiotropic. Taken together, oestrogen plays an important role in prostate carcinogenesis through direct or indirect participation in molecular mechanism which are crucial for tumourgenesis, i.e.: proliferation, cell cycle control, apoptosis or EMT. Further studies are needed to explain all molecular mechanisms of oestrogen signalling in prostate cancer due to their importance in developing new approaches to prostate cancer treatment.

## Abbreviations

2ME2: 2-methoxyoestradiol
ADT: androgen deprivation therapy
AR: androgen receptor
Bax: BCL2-associated X protein
BMI-1: polycomb complex protein BMI-1
CAFs: cancer-associated fibroblasts
CCND1: cyclin D1
c-FLIP: (cellular FLICE (FADD-like IL-1β-converting enzyme)-inhibitory protein
cMyc: v-myc avian myelocytomatosis viral oncogene homolog
CRPC: castrate-resistant prostate cancer cells
DBD: DNA-binding domain
DES: diethylstilbestrol
DHT: 5α-dihydrotestosteron
EGFR: epidermal growth factor receptor
EMT: epithelial–mesenchymal transition
ERs: oestrogen receptors
ERα: oestrogen receptor α
ERβ: oestrogen receptors β
FOS: FBJ murine osteosarcoma viral oncogene homolog
FOXO3: forkhead box O3
FSH: follicle-stimulating hormone
GnRH: gonadotropin-releasing hormone
GSK-3β: glycogen synthase kinase 3
HIF-1α: hypoxia inducible factor 1 alpha subunit
IGFBP-3: insulin-like growth factor-binding protein 3
IL-1β: interleukin-1 beta
IL-6: interleukin-6
iNOS: inducible nitric oxide synthase
iNOS: nitric oxide synthase
JUN: jun proto-oncogene
LBD: ligand-binding domain
LH: luteinizing hormone LH
MAPK: mitogen-activated protein kinases
MIP-2: inflammatory protein 2-alpha
MMP: matrix metalloproteinases
MMP-3: matrix metalloproteinase 3
mTOR: mammalian target of rapamycin
NEAT-1: nuclear paraspeckle assembly transcript 1
NF-κB: nuclear factor kappa-light-chain-enhancer of activated B cells
NR: nuclear receptor superfamily
p21: cyclin-dependent kinase inhibitor 1A
p53: tumour protein p53
PCa: prostate cancer
PHB: prohibitin
PHD2/EGLN1: propyl hydroxylase domain 2
PI3K: phosphoinositide 3-kinase
pRB: retinoblastoma protein
PSA: prostate-specific antigen
PTEN: phosphatase and tensin homolog
PUMA: pro-apoptotic factor p53-upregulated modulator of apoptosis
ROS: reactive-oxygen species
SERMs: selective oestrogen receptor modulators
SGK3: serum/glucocorticoid regulated kinase family member 3
Smad2: SMAD family member 2
Smad3: SMAD family member 3
Smad4: SMAD family member 4
Smad6: SMAD family member 6
Smad7: SMAD family member 7
Snail-1: snail family zinc finger 1
SOX4: sex determing region Y-box
TGF-β1: transforming growth factor beta
TGF-β1: transforming growth factor β1
Thbs 2: thrombospondin 2
Twist-1: twist family bHLH transcription factor 1
UCPs: uncoupling proteins
VEGF: vascular endothelial growth factor
VEGF-A: vascular endothelial growth factor A
VTE: venous thromboembolism
